# Management of a Case of Mucor Colonization in Breast Tissue Expander Seroma Pocket

**Published:** 2018-01

**Authors:** Danielle N Atwood, Pallavi A Kumbla, Brian Yuen, James C Yuen

**Affiliations:** 1 University of Arkansas for Medical Sciences, College of Medicine, Little Rock, AR, USA;; 2 University of Arkansas for Medical Sciences, Department of Surgery, Little Rock, AR, USA;; 3 University of Arkansas for Medical Sciences, Department of Family Medicine, Little Rock, AR, USA;; 4 Banner MD Anderson Cancer Center, Division of Surgical Oncology, Gilbert, AZ, USA

**Keywords:** Mucormycosis, *Mucor*, Breast reconstruction, Expander site infection

## Abstract

Mucormycosis has a mortality rate reaching 90%, and is imperative that therapy be initiated rapidly once a diagnosis is made. Successful treatment consists of management of underlying risk factors, surgical debridement, and antifungal therapies. The dilemma whether or not to pursue extensive debridement presents when the wound is cultured positive but the patient is not systemically ill. We present the first reported case of successful medical treatment of a seroma pocket colonized with mucor in a patient undergoing bilateral reconstruction with tissue expander and acellular dermal matrix.

## INTRODUCTION

Mucormycosis is a rare and potentially life-threatening opportunistic infection with approximately 500 cases per year in the United States.^1^ There are six major presentations, which include rhino-orbital-cerebral, pulmonary, cutaneous, gastrointestinal, disseminated, and uncommon (e.g. osteomyelitis, endocarditis).^2^ Mucormycosis is caused by organisms commonly found in soil and decaying organic matter and is frequently limited to immunocompromised patients such as those with uncontrolled diabetes mellitus.^3^

While rhino-orbital-cerebral and pulmonary presentations are the most common forms of mucormycosis, gastrointestinal, cutaneous, disseminated, and other uncommon presentations have also been reported.^4^^,^^5^ Cutaneous presentations originate from spore inoculation of surgical incisions, traumatic wounds, or burns and initially resemble localized cellulitis, before progressing to deeper infection.^5^ Widespread dissemination of cutaneous forms is due to the extensive angioinvasion characteristic of mucormycosis. Treatment involves correction of underlying risk factors such as hyperglycemia, intravenous or oral antifungal agents, and in cases of severe necrosis, extensive surgical debridement.^4^^,^^6^

The following case is the first reported successful medical management of a mucor-colonized seroma pocket in a patient undergoing immediate staged breast reconstruction following bilateral total skin sparing mastectomies. The patient was treated with oral antifungal agents without loss of the tissue expander and acellular dermal matrix. 

## CASE REPORT

A 41-year old obese (Body mass index: BMI of 31), type II insulin-dependent diabetic female diagnosed with stage II carcinoma of the right breast underwent treatment with neoadjuvant chemotherapy followed by bilateral total skin sparing mastectomies with immediate reconstruction using tissue expanders and acellular dermal matrix (ADM). The ADM employed was ready-to-use AlloDerm (Life Cell Corporation, Branchburg, New Jersey). 

On post-operative day 18, the patient developed bilateral breast seromas, requiring needle aspiration using an 18-gauge IV catheter. Mild erythema was noted at this time and a ten-day course of oral levaquin was started. Aspiration was performed again on the right side two days later. Both seroma cultures grew Mucor (zygomycetes) colonies (few colonies). Oral voriconazole 200 mg every 12 hours was started per infectious disease recommendations. Tight glycemic control (<180) was maintained with close follow-up by her endocrinoloist.

**Fig. 1:**
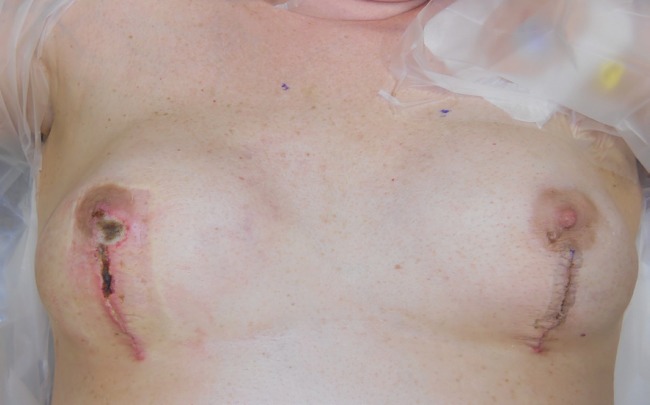
Preoperative photo of bilateral neo breasts with expanders in place immediately prior to excision of necrotic skin on the right side.

**Fig. 2:**
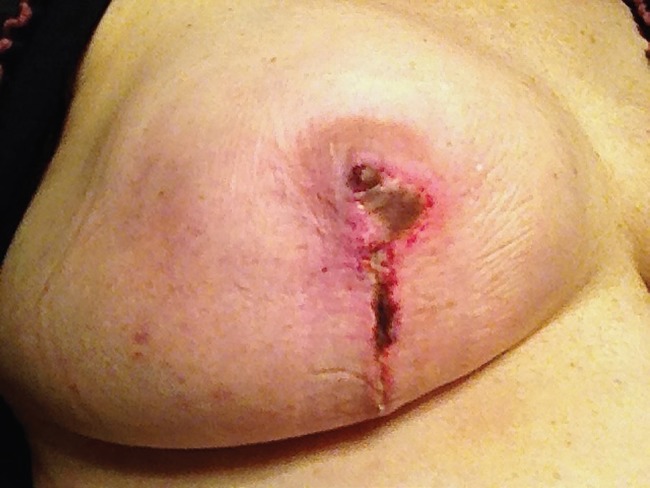
Photos taken three weeks after mastectomy and immediate reconstruction showing development of necrotic skin in the areola and along the incision.

On post-operative day 23, dehiscence was noted at the apex of her vertical incision abutting the areola with an area of skin necrosis from mastectomy skin flap ischemia (Figure 1 and 2). The patient was afebrile and exhibited no signs of sepsis. Debridement of the skin and closure of the wound was performed, preserving the tissue expander and ADM. The ADM was intact without expander exposure. There was no purulence or necrosis of tissue deep to the dermis at the site of dehiscence. Subcutaneous tissue and fluid cultures were positive for Mucor (moderate count). Antifungal therapy was switched to posaconazole 800 mg per oral per day. 

At the time of her drain removal from this second surgery, biofilm strands from the drain site also grew two colonies of Mucor. She required repeat seroma aspiration one week later, and this time the culture was negative. On week later, repeat seroma aspiration grew out skin flora, and this aspiration was followed one day later with abrupt onset of right breast cellulitis which was successfully treated with two weeks of IV ertapenem and vancomycin. She required additional right breast seroma aspirations, two more times over the next 3 weeks. 

The first culture was no growth and second one was positive for skin flora. All aspirations revealed cloudy seroansguinous seroma content, never frankly purulent. Her recurrent seromas resolved, but three months later, her right breast cellulitis recurred, which again responded to IV antibiotics, this time with vancomycin and piperacillin/tazobactam. Thereafter, her healing became uneventful and her tissue expansion was competed to 700 mL bilaterally. 

**Fig. 3:**
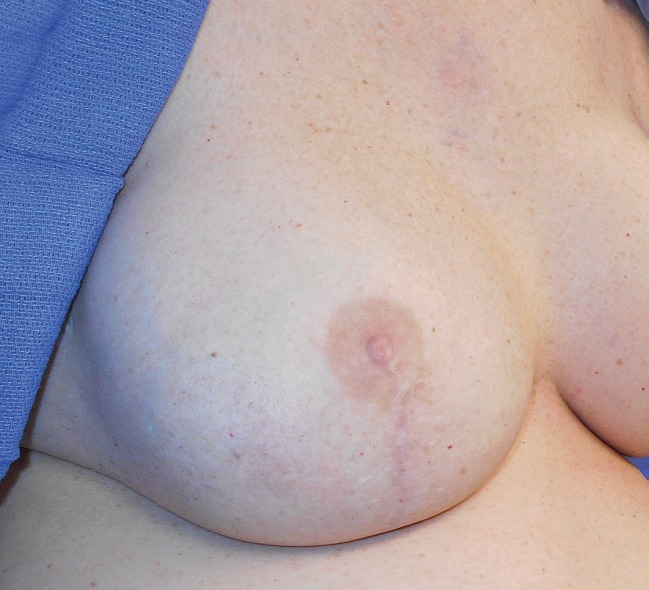
Postoperative photo of the right neo breast 15 months after tissue expander exchange to silicone gel implant.

She completed a one-year course of antifungal under the surveillance of infectious disease consultants, which included close monitoring of liver function tests. Seven weeks after completion of posaconazole, bilateral tissue expander exchange to 750 mL high profile smooth silicone round implants was performed. Capsular tissue appeared normal. Post-operatively two years later, the patient has had no recurrent infections (Figure 3). The patient is currently 3 years after her second-stage breast reconstruction doing well. 

## DISCUSSION

The rate of infection in breast reconstruction ranges from 2.5% to 24%, and with the increasing volume of breast reconstruction post-mastectomy in the United States,^7^ this serious and potentially life-threatening complication warrants attention. The patient described above had multiple risk factors for perioperative infection, including previous chemotherapy, diabetes mellitus, and a BMI >25.^7^ Bacterial infection is a well-known cause for surgical site infection, but there currently exists no reported cases of mucormycosis associated with breast reconstruction. While fungal tissue infections of tissue expanders has been reported,^8^^-^^10^ none of the reported cases involved cutaneous mucormycosis. 

Cutaneous Mucor infection can quickly become locally invasive, spreading to adjacent fascia, muscle, bone, vasculature, and ultimately causing necrotizing fasciitis and wide dissemination. The mortality rate for Mucor necrotizing fasciitis and disseminated mucormycosis is 80% and >90%, respectively.^1^ Due to the rarity of this infection, there is a lack of clinical trials regarding appropriate antifungal therapy in mucormycosis, thereby leaving a large dearth of information for clinicians attempting to treat these infections. There is also no standard surgical protocol in the management of a tissue expanders seroma pocket which is colonized with Mucor. 

While the patient was not systemically ill and was free of necrotizing soft-tissue infection, the best practice question was raised whether to remove the acellular dermal matrix and tissue expander and leave her with a major deformity. We are fortunate to report the successful use of posaconazole, which has known activity against the fungus,^1^ without loss of the her breast reconstruction. Because of the known fact that mucormycosis in the diabetic patient is potentially lethal, it became a conundrum in decision-making of what to do with the tissue expander and ADM once the seroma culture came back positive for Mucor species. 

This patient had low-grade erythema at the time, but she was free of systemic signs of sepsis; therefore, explantation was not entertained. The decision became increasingly difficult when she presented later with dehiscence, which was felt more likely secondary to her mastectomy flap ischemia. Possible loss of her breast reconstruction was posed again when she developed two episodes of right breast cellulitis, but both times the cellulitis rapidly responded to IV broad-spectrum antibiotics. The decision against explantation was based on the rapid resolution of erythema following the administration of IV antibiotics and the absence of sepsis. 

Since the management of positive cultures for Mucor related to tissue expander in breast reconstruction has never been reported, this case report provides valuable information to determine treatment pathway for such a rare clinical presentation. In the absence of wound and systemic sepsis, Mucor colonization of the expander seroma pocket was treated medically. Prompt recognition and intervention with appropriate antifungal therapy played a favorable role in resolving this Mucor colonization without loss of breast reconstruction. 

The presentation and course of treatment in this case of Mucor colonization of a breast tissue expander pocket have not been previously reported in the English literature. While this case had a favorable outcome, the surgeon must be vigilant in the follow-up care of such patient. The senior author has had more than 20 years of experience in distinguishing the difference between a necrotizing infection versus a case of low-grade infection or colonization. When it doubt, the tissue expander and acellular dermal matrix should be removed, especially if there is any sign of systemic illness. Any sign of necrotizing fasciitis would also require aggressive debridement. 

Retrospectively, the Mucor species presented in this case proved to be non-invasive. Had the colonization of Mucor advanced to cutaneous mucormycosis, the treatment course would have ended with radical surgical debridement for life-saving measure and secondary major deformity. Extreme caution and compulsive follow-up care are needed when medically treating a patient with a seroma or wound perceived to be colonized with Mucor. Any progression towards soft-tissue necrosis warrants rapid return to the operating room. 
